# The equation for medical multiple-choice question testing time estimation

**DOI:** 10.1097/MS9.0000000000002010

**Published:** 2024-04-04

**Authors:** Chatchai Kreepala, Nattawut Keeratibharat, Sekdusit Aekgawong, Krittanont Wattanavaekin, Taechasit Danjittrong, Thitikorn Juntararuangtong, Theetad Chombandit

**Affiliations:** a School of Internal Medicine; b School of Surgery, Institute of Medicine, Suranaree University of Technology, Nakhon Ratchasima; c Department of Surgery, Faculty of Medicine, Ramathibodi hospital, Mahidol University, Bangkok; d Department of Anesthesiology, Chulabhorn Hospital, Bangkok, Thailand

**Keywords:** MCQ, medical assessment, testing time estimation

## Abstract

**Introduction and importance:**

Multiple-choice questions (MCQs) offer a suitable means to assess the cognitive domain of learners with a high degree of objectivity. The study's objective is to formulate an equation for determining the ideal timing for MCQ examinations, thereby inspiring the development of a model to estimate the duration of these examinations.

**Methods:**

The authors generated a specific computer program that integrated with the operating system of the examination. Technical-specific features included the ability to calculate the speed of students taking examinations with images or videos in the questions or options. This bespoke computer program was designed specifically for assessing individual students' MCQ test-taking pace and generating a proctor report in a computer-readable format. Subsequently, data derived from this program underwent regression analysis to determine the speed at which students completed MCQ examinations.

**Outcomes:**

The data were collected from a total of 1035 examinees, all of whom were non-native English speakers. The average reading rate was 62.38±20.4 words/min. It was found that the rate decreased significantly in difficult (50.65±6.9 words/min) items compared to easy (82.29±21.3 words/min) and intermediate (60.56±19.1 words/min) items (*p*<0.001), respectively. The linear regression analysis predicted option selection (words/min) as; 33.92+1.93(%tables/figures)+0.14(%recall)–0.37(%application), r^2^=0.45, *p*<0.001.

**Conclusion:**

It is not advisable to base the decision solely on reading time or time allocation. Examination administrators are advised to proactively plan ahead, with particular emphasis on establishing a well-defined taxonomy, as it constitutes a fundamental cornerstone in the utilization of the estimation equation.

## Introduction

Highlights

The multiple-choice question (MCQ) is one of the objective assessment methods. It is proper to evaluate the cognitive domain of learners with high objectivity. However, there are no definite criteria for determining the appropriate examination time. This paper would demonstrate….

- The criteria for determining the appropriate examination time.
- The improvement of the learning assessment in medical school.
- The equation of examination time is based on the ability to comprehend the meaning of the text.

Multiple choice questions (MCQs) are the most widely used assessment method, especially in medical school^1–7^. In addition, it is also easier to create when compared to other assessments in terms of content validity, uncomplicated preparation, and suitability for large amounts of students^8–11^.

Nevertheless, determining the appropriate testing time for MCQ examinations is not a straightforward task. From previous reports, it was found that the average college student (native English) reads 230–250 words per min, a reading speed impossible for comprehension purposes, particularly among non-English speakers^12,13^. The author’s prior sequential research aimed to explore factors impacting testing duration through a qualitative study^14^. The study revealed five constructive domains: (1) the number and total word count, (2) positive factors of difficulty (application/calculation questions), (3) negative factors of difficulty (recall questions), (4) examiners (native English speakers or not), and (5) pictures/symbols in the tests.

While Bloom’s taxonomy typically determines the learning level of widely used test items, establishing a framework for measuring outcomes, such as knowledge, understanding, application, analysis, synthesis, and evaluation^15^. However, MCQs employed in graduate medical degree assessments often exhibit fewer instances of analysis, synthesis, and evaluation questions. Therefore, in this study, analysis, synthesis, and evaluation questions were incorporated into the category of application/calculation questions to calculate an appropriate test duration equation.

The insights accumulated from this foundational grounded study provided fundamental data for this ongoing research. This research involved an analysis encompassing difficulty levels of the MCQs, English-MCQs reading rates by non-native English speakers, and the proportion of students who completed or finished too quickly. Leveraging earlier qualitative data on examination question difficulty^16^, an equation was devised to ascertain an optimal testing duration, serving as the principal objective of this study.

## Methods

### Data sources

The study was conducted at the Institute of Medicine, Suranaree University of Technology in Thailand. The research participants were medical students in both preclinical and clinical years between the academic years 2021 and 2022. The MCQ examinations in this study were in the format of a single best response, which consisted of a question followed by five possible answers.

### Eligibility criteria

All examinations in this study were computer-based, closed book, single best answer MCQs written in English. All of the participants were non-native English speakers which were defined as those students who spoke a language other than English at home^17–19^.

### Interventions

The researchers developed a specialized software program that integrated seamlessly with the operating system of the medical school examination system to assess the timing of multiple-choice question (MCQ) tests. This program was capable of generating comprehensive reports and proctor reports for all courses employing MCQ examinations, both for individual students and entire courses, within the web-based examination system (Exam Plus program)^19^ Additionally, it had the capability to access predefined examination parameters such as recall, comprehension, and application questions^15^. Noteworthy technical functionalities included the ability to calculate students’ examination-taking speed and to gauge the pace of individual students when responding to MCQs containing images or videos in the questions or options. This research was conducted in accordance with the SQUIRE-EDU criteria^20^.

### Participants and comparators

In this study, the participants were divided into three groups for data analysis:

Group A (incompleted) consisted of students who were unable to complete the test within the given time duration and were further subdivided as follows:

Group A1 (unfinished) consisted of students who were unable to complete the test on time. There were some questions left unanswered at the end of the examination.

Group A2 (guessing) constituted students supplied random answers to unanswered questions. This behaviour was discerned through an observed surge in completion speed exceeding 200% after the 5-minute time notification before the end of the examination. This phenomenon suggested a pattern indicative of guessing.

Group B (rapidly completed) consisted of students who completed the MCQ test before 50% of the allocated test time had elapsed for the subject without speeding up answering.

Group C (completed) consisted of students who completed the MCQ test after 50% of the allocated test time had elapsed for the subject, without suspicious speeding up of answering.

### Outcomes

The researchers aimed to determine the appropriate time duration for single best answer MCQ examinations using logistic regression analysis to predict whether the examination would be completed on time. This was done to establish an equation for time allocation, with the assumption that an appropriate examination duration would have enabled students to answer more than 80% of the questions based on their knowledge rather than guessing due to time constraints.

### Included and excluded studies

The MCQ examination utilized in this research must adhere to a single best-answer format. The examination should consist solely of MCQs, and all questions must be presented in English. The examination administration must be conducted as a computer-based test. Cases where examinations include questions in languages other than English or incorporate other assessment tools, including online examinations allowing students to complete them outside the examination room, are to be excluded from this study.

### Study selection

During the preclinical years, the study encompassed three learning organ systems (haematology, urinary, and gastrointestinal system). Additionally, six clinical learning subjects from two major departments, Medicine and Surgery, were incorporated. Medical students from another academic year who were retaking the examination in 2021–2022 were also included. The formative examination was omitted from this study.

### Data collection process

Regarding the analytical functionality of this program, it was capable of determining the pace of each student during MCQ examinations. This was achieved by indicating the number of questions answered by each student when 50% of the examination time had elapsed, when 25% of the examination time remained, during the final 15 min, the last 5 min, and at the conclusion of the examinations. The evaluation of MCQ timing was subjected to linear regression analysis to identify significant influencing factors and establish a predictive equation for determining the appropriate test duration. The system possessed the ability to assess the examination speed of individual students and could generate a proctor report in a computer-based format.

### Risk of bias

The study conducted a grounded study on factors previously identified^14^. However, within the same demographic group, there might have been biases from prior studies, such as recall bias, where students may remember the characteristics of the examinations, their difficulty level, or not all questions. The researchers addressed this concern by randomizing inquiries during various examination periods in previous studies and ensuring saturated data from qualitative data collected in prior studies^14^.

### Synthesis of results

In the study, researchers utilized computer systems to calculate examination durations, distinguishing between students who had completed their examinations before the scheduled time and those who had not. Subsequently, the obtained examination durations were evaluated alongside the characteristics of the questions—recall, comprehension, and application—to delineate their levels of difficulty. Following this analysis, logistic regression analysis was employed to forecast whether examinees would complete the examination within the designated time frame without resorting to guessing. Furthermore, the appropriate examination duration was determined, taking into account the weighting factors incorporated into the equation.

### Statistical analysis

Statistical analyses were performed for quantitative analysis with SPSS Statistics for Windows^21^. The differences between the two groups were compared using the Student’s *t*-test, and the χ^2^ test for the continuous and categorical variables, respectively. To establish a suitable equation for predicting examination time, the researcher employed statistical linear regression analysis to determine the significant factors that correlate with the examination rate. *P* values less than 0.05 were considered statistically significant.

## Results

### Demographic information

The data were collected from a total of 1035 examinees, all of whom were non-native English speakers. There were 329 male examinees (31.8%) and 706 female examinees (68.2%). The average age was 21.88±1.4 years, the average grade was 3.55±0.4, and the average reading rate was 0.96±0.3 questions/min or 62.38±20.4 words/min (word count).

The detailed summative MCQ tests consisted of 10 tests in preclinical medical science (year 2–3) and 10 tests in clinical medical science (year 4–5). Each set of MCQ tests had an average of 93.86±8.9 items per test (range: 80–100 items per test) and an average word count of 6139.12±1434.3 texts per test (range: 4007–8490 texts per test), or an average of 64.88±11.4 texts per item. The tests contained an average of 16.57±6.1% (range: 11–34%) questions with images or tables and had an average duration of 120.91±22.4 minutes per test (range: 100–180 min per test), with an average of 66.94±27.3 students per test (range: 13–94 students per test).

When the MCQ examinations were classified into three levels of difficulty, it was found that there were 4/20 easy level MCQ tests, with a total of 185 students (17.9%) taking the tests; 10/20 intermediate level MCQ tests, with a total of 635 students (61.4%) taking the tests; and 6/20 difficult level MCQ tests, with a total of 215 students (20.8%) taking the tests.

### Decision-making in selecting answers and guessing

**Figure 1:**
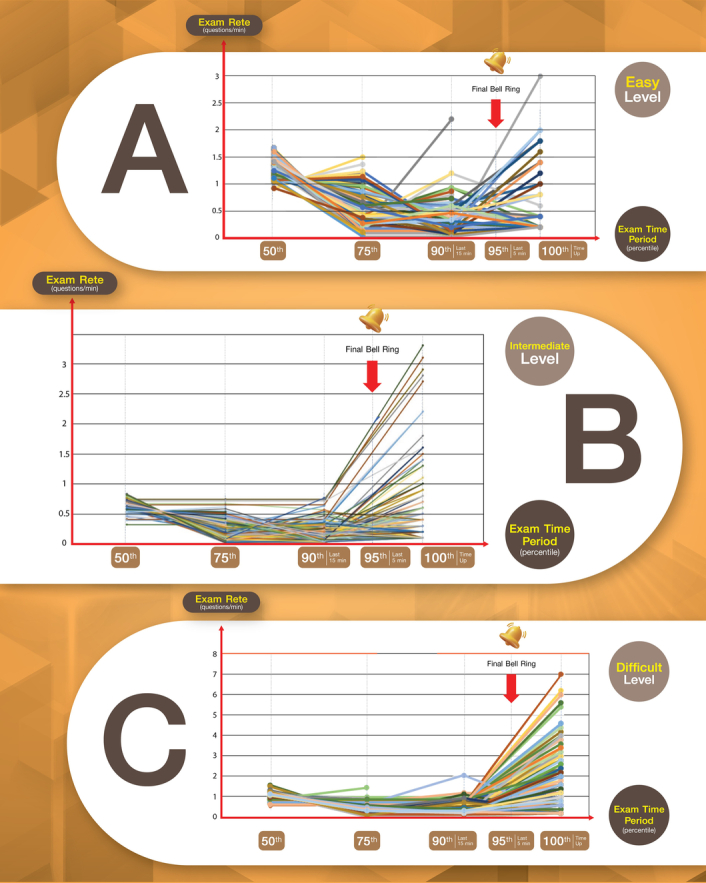
The exam speed trend at five different periods of examination; 50–75 percentile, 75–90 percentile, 90–95 percentile (last 15 min of test time) and 95–100 percentile (after bell ring or last 5 min of test time). The trend of different difficulty levels were shown as picture (A) easy, (B) intermediate, and (C) difficult.

The researchers developed a program that monitored the behaviour of students during English, computer-based, closed-book MCQ examinations. The program recorded the number of questions the students answered during different time intervals, including the end of the first 50th percentile, at 75th, 90th, and 95th percentiles, and the end of the examination (100th percentile). The program signalled the end of the examination with a bell ring, which occurred 15 and 5 min before the end of the examination and was close to the 90th and 95th percentiles of the examination duration. The program then calculated the average rate of answering questions for each student at each time interval. A trend line was drawn through these data points to show the trend of each student’s average rate of answering questions in four time intervals: 50–75, 75–90, 90–95, and 95–100 percentiles of the examination duration. Additionally, the program recorded which students completed the examination without any additional changes, as well as the students who did not finish the examination and the number of questions remaining (Fig. 1).

The details in Fig. 1 showed that for the examination with various difficulty levels, the easy level (a set of questions with a recall question rate of more than 65% of the total questions) allowed students to complete the examination quickly in the early and middle stages. However, they might reconsider their answers towards the end of the examination (A). On the other hand, for the intermediate (B) and difficult levels (C), completing the examination in the early stages was slow, but the pace significantly increased when the time was about to expire. Some students, in this case, may not finish some questions, prompting them to speed up to complete them before the examination time runs out. The author assumed that there might have been guesswork involved during this time.

According to the data, students tended to perform quickly on examinations during the initial 50th percentile. However, their rate of performance decreased to a steady level during the 50–75th percentile and 75–90th percentile, respectively. Furthermore, the rate of performance increased significantly after the last 5-min warning signal before the end of the examination.

The analysis data on the difficulty levels of the examination revealed that students exhibited consistent test-taking behaviour regardless of the examination’s difficulty level. The researcher analyzed the average rate of examination completion for the entire group of students, from the start of the examination until 15 min before the examination’s end or until the completion of the examination (in the case of students who finished before the end time). The mean difference between the average rate of examination completion during the last 5 min and the overall average rate of examination completion was 38.9% (95% CI; 28–48%). Therefore, students who completed the examination within the last 5 min with a rate of completion greater than 40% of the overall average rate of completion were considered to guess on some questions due to time constraints.

To classify students based on their exam-taking behaviour, the researcher grouped students into three categories: A: incompleted, B: rapidly completed, and C: completed. The students who guessed on some questions due to time constraints were classified as “Rapidly Completed” students.

**Figure 2:**
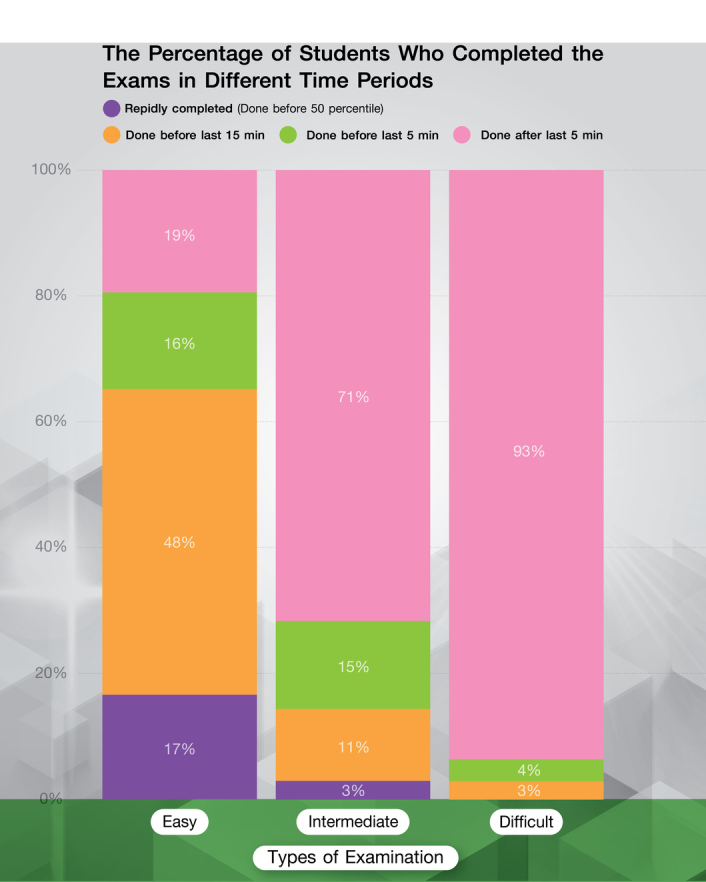
The proportion of completed examination students; The bar chart depicted the completion times of students who took an exam at different difficulty levels. The chart revealed that in examinations with an easy level of difficulty, a significantly larger number of students completed the examination rapidly compared to examinations with higher levels of difficulty, where this trend was not observed. On the other hand, it was found that as the difficulty level of the exam increased, students required longer completion times to finish the examination. (χ^2^ *P* < 0.001).

When the examination was divided into three levels based on its difficulty level (i.e. easy, intermediate, and difficult) and analyzed alongside the rapidly completed group, it was found that the rapidly completed group had a higher incidence rate on the easy level examinations (17%) compared to the other groups, and this group was not found in the difficult level examinations. Additionally, students who completed the examination within the set time limit had a higher incidence rate of correct answers compared to the rapidly completed group (*P* <0.001) (Fig. 2). The proportion of completed examination students; The bar chart depicted the completion times of students who took an examination at different difficulty levels. The chart revealed that in examinations with an easy level of difficulty, a significantly larger number of students completed the examination rapidly compared to examinations with higher levels of difficulty, where this trend was not observed. On the other hand, it was found that as the difficulty level of the examination increased, students required longer completion times to finish the examination.

### The reading speed of English examination questions and the decision-making process

It was observed that as the difficulty of the examinations increased, the proportion of students who completed the examination decreased sequentially when comparing easy to difficult levels (78.9%:78.1%:53%, *P*<0.001).

The reading speed of English examination questions and the decision-making process for selecting answers were analyzed based on the average examination completion rate. The analysis included the number of texts (word count), demographic characteristics of participating students, as well as the number of students who completed or did not complete the examinations in each test. The analysis was conducted according to the difficulty level of the MCQ (Table 1).

**Table 1:** Evaluating factors affecting MCQ test time among the various difficult examinations

	The difficulty of MCQ Level					
Factor affecting on the MCQ test time	(A) Easy	(B) Intermediate	© Difficult			*P*
Tests’ Factors						
Test Time (min) [mean (min-max)]	126.49 (120–180)	124.57 (100–180)	105.30 (100–120)	A vs. B 0.582	A vs. C <0.001	B vs. C <0.001
Question number per Test (n) [mean (min-max)]	100 (100–100)	95.87 (80–100)	82.65 (80–90)	A vs. B <0.001	A vs. C <0.001	B vs. C <0.001
Word count per Test (*n*) [mean (min–max)]	6788.99 (5425–8490)	6264.61 (4007–8490)	5209.27 (4823–6280)	A vs. B <0.001	A vs. C <0.001	B vs. C <0.001
Word count per Question (*n*) [mean (min–max)]	67.89 (54.3–84.9)	64.71 (50.1–84.9)	62.80 (60.3–69.8)	A vs. B 0.013	A vs. C <0.001	B vs. C 0.002
Question rate (mean±SD, questions/min)	1.23±0.3	0.93±0.2	0.81±0.1	A vs. B <0.001	A vs. C <0.001	B vs. C <0.001
Word reading speed (mean±SD, words/min)	82.29±21.3	60.56±19.1	50.65±6.9	A vs. B <0.001	A vs. C <0.001	B vs. C <0.001

max, maximum; MCQ, multiple-choice question; min, minimum; *n*, number.

When considering the characteristics of the examinations, which were designed by the examiners, it was found that examinations with greater difficulty were given less time compared to easier and moderate examinations, respectively (*P*<0.001). This indicated that examiners considered the level of difficulty when designing the examination and reduced the number of examination questions or used shorter test items if the examination was considered difficult.

In terms of the average rate of answering and reading speed, it was found that this rate decreased significantly in difficult (50.65±6.9 words/min) examinations compared to easy (82.29±21.3 words/min) and intermediate (60.56±19.1 words/min) examinations (*P*<0.001). However, the proportion of students who completed, did not complete, or completed the examination quickly varied across different levels of examination difficulty (Table 1).

### The predictive formula for the determination of examination time

To establish a suitable equation for predicting examination time, the researcher employed statistical linear regression analysis to determine the significant factors that correlated with the examination rate. The analysis revealed three factors that had a significant correlation with examination rate (words/min) at *P* less than 0.001 without any evidence of spurious correlation resulting from multicollinearity and hence adopted them into the equation. It should also be further noted that all factors had a Variance Inflation Factor value not exceeding 4.0 and a Collinearity tolerance not less than 0.2, as shown in Table 2.

**Table 2:** Linear Regression analysis (stepwise method) of factors affecting examination time for developing the estimation examination rate formula

	Multivariate coefficients		95% CI for beta			Collinearity statistics	
	Beta	SE.	Lower bound	Upper bound	*P*	Tolerance	VIF
(Constant)	33.924	3.582	26.896	40.953	<0.001		
Proportion of question with tables and figures (%)	1.930	0.097	1.740	2.119	<0.001	0.635	1.576
Proportion of recall questions (%)	0.141	0.028	0.086	0.196	<0.001	0.339	2.952
Proportion of application questions (%)	-0.369	0.058	-0.483	-0.255	<0.001	0.382	2.621

SE, standard error of the regression, VIF, variance inflation factor.

After utilizing the data presented in Table 2 to develop a linear equation, the correlation employed in predicting examination time was established. The prediction equation was derived in the form of an examination rate, which was the ratio between the word count of the entire examination and the time taken (in min), rounded to two decimal places (Equation 1). If there was a desire to compute the examination time, the word count of the actual examination must be multiplied by the reciprocal of this ratio (Equation 2).


$$Words/Min=33.924+1.93\;(\%tables+figures)+0.141\;(\%recall)-0.369\;(\%apply)r^{2}=0.50,P<0.001$$

[1] Words/Min=33.92+1.93(%tables+figures)+0.14(%recall)−0.37(%apply)

[2] $$\mathrm{So, the estimated exam time}=\mathrm{Word count}\times \frac{1}{\left[33.92+1.93\;(\%tables+figures)+0.14\;(\%recall)-0.37\;\left(\%apply\right)\right]}$$

## Discussion

Based on the data, it was suggested that teachers would consider reducing the number of test items and text length in each item if they determined that a given test set was more difficult than others instead of increasing the examination duration. This approach may help alleviate the significant occurrence of incomplete responses among test takers, particularly within the “incomplete” subgroup. When comparing difficult levels with the “easy” and “intermediate” test sets, this consideration supported the notion of adjusting the examination duration by the varying levels of difficulty across test items.

It was found that the average reading rate for students during examinations was 0.96±0.3 questions/min, which translated to an average word count of 62.38±20.4 words/min. The rate of reading during difficult examinations was slower at 0.81±0.1 questions/min compared to easy examinations at 1.23±0.3 questions/min and intermediate at 0.93±0.2 questions/min, with statistical significance at *P* less than 0.001. This behaviour could lead to reduced validity of the examination due to construct underrepresentation, as the measure did not fully cover the intended construct^22^. However, no matter how much time the teacher gave for the examination, and how difficult it was, in the end, students would try to guess to get a chance to score. This corresponded to the finding that guessing and fast rates of answering increased when a warning signal was given 5 min before the end of the examination^23^.

This information was consistent with the previous study conducted by the research team themselves^13^, which emphasized that reading for comprehension differs from reading for decision-making^12,13^. It was not appropriate to use reading rates for comprehension as a determinant of reading time for decision-making^12^. It should be noted that this report was the first to present reading rates specifically for decision-making tasks, particularly for non-native English speakers who were Thai^23^.

It should be noted that word count or question count alone might not be sufficient as measures, as examination difficulty may also affect the required decision-making time. In practical medical settings, it is essential to gather enough information to support decision-making, diagnosis, or problem-solving, as insufficient time for decision-making may lead to errors. Thus, MCQ examinations might not be able to fully replicate real-life scenarios^25^.

The National Board of Medical Examiners suggests that each MCQ should ideally be given 60–90 sec for decision-making reading time^26–28^. However, the predetermined time frames mentioned above may not be suitable for each examination because the difficulty levels of the questions vary. This study provided clear evidence that a significant number of students rapidly completed examinations in the easy-level question group, while students in the difficult-level question group were not found to complete examinations quickly. This indicated that the difficulty level of the questions greatly affected the decision-making time.

Correlation coefficient and linear regression analyses indicated that factors influencing examination duration from the students’ perspective, besides English proficiency and content coverage, also included the taxonomy of the test, which affected the level of difficulty of the test items.

## Conclusion

Setting an appropriate examination time for MCQs did not involve a simple consolidation. The requirement for determining the appropriate examination duration for MCQs had never been studied. Examinations typically adhered to traditional time limits, often arbitrarily set by the examiners. This report suggested that if examinations were to truly measure students’ knowledge, it was advisable to establish a clear definition of examination duration based on calculations derived from the equations developed in this research. For example, allocating 60 min for 50 questions, as previously practiced, was not recommended. It was also not advisable to use reading time as a determining factor for the rate or duration of completing the examination. Instead, considerations should be made based on the length of the questions, the difficulty level of the examination, and the inclusion of tables or images in the questions. Most importantly, the taxonomy (recall, comprehension, application) must be defined for each question before applying the examination time equation. If there are inaccuracies in determining the taxonomy, it will affect the accuracy of the estimation equation.

## Ethical approval

The participation protocol was approved by the Human Research Ethics Committee, Suranaree University of Technology (Issue # EC-64-102).

## Consent

The authors disclosed to these patients whether any potential identifiable material might be available via the Internet as well as in print after publication. Nonessential identifying details were omitted and replaced by special research codes. Informed consents were obtained if there is any doubt that anonymity can be maintained. Written informed consent was obtained from the participants for publication and any accompanying images. A copy of the written consent is available for review by the Editor-in-Chief of this journal on request.

## Source of funding

This work was supported by the Grant of Suranaree University of Technology (contract number SUT-602-64-12-08(NEW)).

## Author contribution

C.K. conceived of the presented idea, developed the theory, and performed the computations and discussed the results and contributed to the final manuscript. N.K., S.A., K.W., and T.D. discussed the results and wrote the manuscript with support from C.K. T.C. and T.J. designed the model and the computational framework and analyzed the data.

## Conflicts of interest disclosure

None of the authors have a conflict of interest to disclose.

## Research registration unique identifying number (UIN)

https://www.clinicaltrials.gov/. NCT06210997.

## Guarantor

Assoc. Prof. Chatchai Kreepala.

## Data availability statement

The datasets generated during the current study are publicly available, available upon reasonable request.

## Provenance and peer review

Not commissioned, externally peer-reviewed.
